# Excellent results of screening for subsequent breast cancers in long-term survivors of childhood Hodgkin's lymphoma—Results of a population-based study

**DOI:** 10.3389/fped.2023.1161128

**Published:** 2023-04-03

**Authors:** Lorna Zadravec Zaletel, Maja Cesen Mazic, Janez Jazbec, Gregor Kos, Miha Toplak, Danijela Štrbac

**Affiliations:** ^1^ Department of Radiotherapy, Institute of Oncology Ljubljana, Ljubljana, Slovenia; ^2^ Faculty of Medicine, University of Ljubljana, Ljubljana, Slovenia; ^3^ Department of Oncology and Haematology, University Children’s Hospital, University Medical Centre, Ljubljana, Slovenia

**Keywords:** subsequent breast cancer, breast screening, childhood cancer survivor, chest irradiation, long-term follow-up, hodgkin' lymphoma

## Abstract

**Introduction:**

Subsequent breast cancer (SBC) represents a major complication in childhood cancer survivors and screening for SBC in survivors after incidental irradiation of breasts is recommended. In this article, we report the results and discuss benefits of SBC screening in female pts treated for Hodgkin's lymphoma (HL) in Slovenia in a period of 45 years.

**Methods:**

Between 1966 and 2010, 117 females were treated for HL under the age of 19 in Slovenia. One hundred five of them survived for 5 years and were included in our study. They were 3–18 (med. 15) years old at diagnosis and followed for 6–52 (med. 28) years. Eighty-three percent of them had chest RT with a median dose of 30 Gy. Ninety-seven (92%) of 105 pts were regularly followed according to the international guidelines including yearly screening mammography/breast MRI in those who received chest RT.

**Results:**

We diagnosed 10 SBCs in eight pts 14–39 (med. 24) years after diagnosis at the age of 28–52 (med. 42) years. At 40 years of follow-up, cumulative incidence of SBCs in females who got chest RT was 15.2%. Seven of eight patients (with 9 SBCs) got chest RT with 24–80 (med. 36) Gy at the age of 12 to 18 (median 17) years. Two patients in this group got bilateral SBC. One patient got invasive SBC after being treated with ChT containing high-dose of anthracyclines without chest RT at the age of 13. All eight invasive SBCs were invasive ductal cancers, HER2 receptors negative, all but one with positive hormonal receptors. Six invasive cancers were of stage T1N0, one T1N1mi, only one, diagnosed before era of screening, was of T2N1. None of 8 pts died of SBC.

**Conclusion:**

After introduction of regular breast screening in our female patients, who received chest RT in childhood, all SBCs were of early stage and no patients died of SBC. Survivors of pediatric HL should be informed about the risk of late sequelae of treatment for HL, including SBC. Regular follow-up with breast cancer screening and breast self-examination is of vital importance in those treated with chest RT.

## Introduction

Survival of children with cancer improved considerably (1). In Slovenia, 86% of children with cancer diagnosed in the last decade became long-term survivors (2). However, approximately 75% of childhood cancer survivors (CCS) will have at least one chronic health condition during adulthood (3). One of the most devastating sequelae among CCSs is the occurrence of subsequent primary neoplasms, with risks ranging from three- to six fold that expected (4–6). Subsequent breast cancers (SBC) are among the most frequent subsequent primary neoplasms (4–6). Radiation therapy is an established risk factor for SBC among survivors of a childhood or young adult cancer (3). Higher doses of radiation to breasts, larger field size, and younger age at exposure, increase SBC risk (7–13). The cumulative risk of SBC is around 35% at 40 years of age following mediastinal irradiation, which is 75 times the risk in the general population (7). This risk is similar to that in the high-risk population that present with predisposing BRCA1 or BRCA2 germ-line mutations (14, 15) and is substantially higher than that in young women in the general population, in whom the cumulative incidence of invasive breast cancer by age 45 years is only 1% (16). Henderson et. al investigated SBC risk in CCS without a history of chest radiotherapy (RT) and found out that alkylating agents (AA) and anthracycline containing chemotherapy (ChT) were associated with increased SBC risk in a dose-dependent manner (17). Similarly, Ehrhardt et al. reported that higher doses of anthracyclines are associated with increased risk of SBC in CCS and is possibly mediated by TP53 mutation-related gene-environment interactions. They observed a greater than 13-fold risk for breast cancer in women exposed to 250 mg/m^2^ or more of anthracyclines compared with none (18).

Specialized follow-up with breast screening is recommended for women at high risk of SBC in order to detect them early and to optimize their management. According to consensus guidelines there are recommendations for annual risk-based breast cancer surveillance with mammography or breast MRI or a combination of mammography and MRI for women exposed to 20 Gy or more of chest radiation, beginning at 25 years of age or 8 years or more after exposure, whichever occurred later (19). Surveillance imaging identifies SBC less likely to require ChT than those detected by physical findings.

In Slovenia, long-term follow-up clinic for CCS from whole country was established in early 1986 (20) at the Institute of Oncology. In 1999, we introduced regular annual screening for SBC in females who got chest RT as part of cancer treatment. At yearly checkups, beside physical examination and referral for screening mammography/breast MRI, patients are referred for other needed examinations (cardiac, endocrinological…) according to international guidelines depending on type of treatment. In this article, we report the incidence, histological properties and survival of SBC in female pts treated for Hodgkin's lymphoma (HL) in Slovenia in a period of 45 years and discuss benefits of breast screening.

## Material and methods

Between 1966 and 2010, 117 females were treated for HL under the age of 19 in Slovenia. Medical events, treatment exposures, and vital status were abstracted from medical reports and cancer registry follow-up. The primary outcome was development of a SBC (invasive and *in situ* carcinomas). Records were obtained, and breast cancer characteristics were abstracted including histology, diagnosis date, age at diagnosis, detection method (physical findings by survivor or provider, imaging), size, nodal involvement, hormone receptor status, HER status, intervention (surgery, ChT, hormone therapy, and/or radiation).

In December 2022, 92 female patients were alive. Twenty-five female patients died, twelve less than 5 years after diagnosis. One hundred five 5-years survivors were included in our study. Eight (7.5%) pts were lost to follow-up, but we got data about possible subsequent malignancies and vital status of these pts from our cancer registry. Ninety-seven pts were regularly followed according to the international guidelines (19, 21); 91 pts at the outpatient department for long-term follow-up at our Institute of Oncology and six pts in cancer centers abroad according to our recommendations. We perform yearly screening mammography (sometimes with ultrasound) in females who received chest RT starting at 25 years of age (8 years after chest RT), for the last 15 years we alternate yearly mammography with breast MRI. One hundred-five patients were 3–18 (med. 15) years old at diagnosis and followed for 6–52 (med. 28) years. Eighty-seven of them had chest RT with a total dose of 20–80 (med. 30) Gy in 1.5–2 Gy per fraction. One patient was treated on orthovoltage machine; others were treated on cobalt 60 machine or linear accelerator. Sixty-seven (77%) pts who got chest RT received ChT as well, all but one with AA, 51 of them anthracycline containing ChT. Eighteen patients had no chest RT, 12 of them got ChT, 12 with AA, nine anthracycline containing ChT (Table 1).

**Table 1:** Characteristics of 105 5-years survivors of HL included in the study.

	Patients	
	Number (*N* = 105)	Percent (%)
Age at diagnosis of HL (years)	3–18 (med. 15)	
Follow—up time (years)	6–52 (med. 28)	
**Treatment for HL**		
Type of treatment		
Chest RT and ChT	67	77
Chest RT, no ChT	20	19
ChT and no chest RT	12	11
No ChT and no chest RT	6	6
RT dose to the breast tissue in 87 pts who had chest RT (Gy)	20–80 (med. 30)	

RT, irradiation; HL, Hodgkin’ lymphoma; ChT, chemotherapy.

We used the Kaplan-Meier method to estimate cumulative incidence of SBC in a whole cohort and separately for the group of females treated with chest RT and those who did not receive chest RT.

### Ethical considerations

The study was reviewed and approved by institutional ethics commission (ERIDEK-9978/2022).

## Results

**Figure 1:**
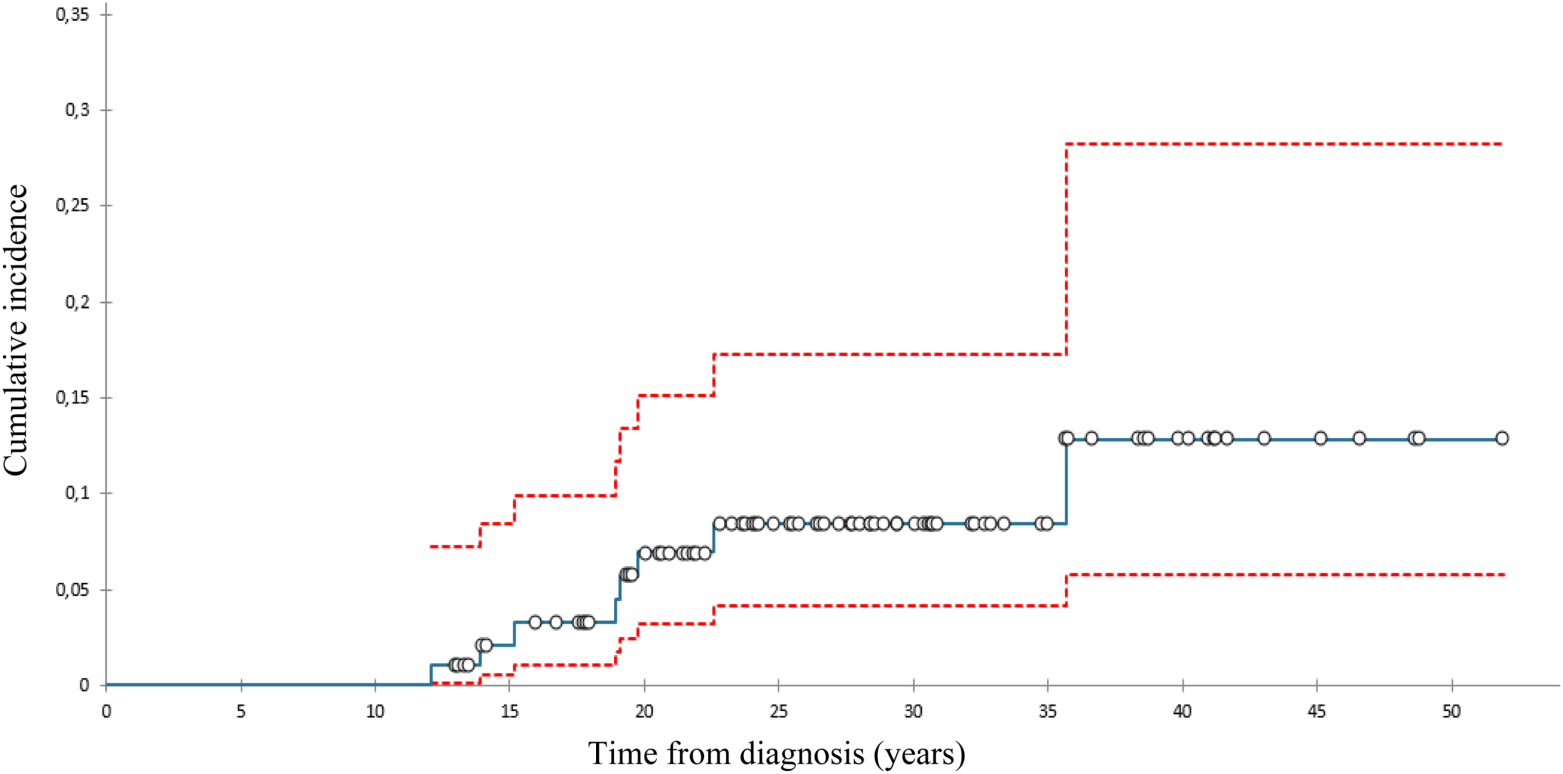
Cumulative incidence of subsequent breast cancer (with 95% confidence interval), all patients.

**Figure 2:**
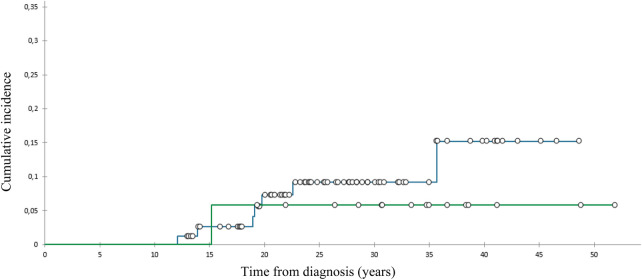
Cumulative incidence of subsequent breast cancer, patients with (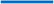) and without (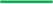) chest radiotherapy.

We diagnosed 10 SBCs in eight pts 14–39 (med. 24) years after diagnosis at the age of 28–52 (med. 42) (Table 2). At 40 years of follow-up, cumulative incidence of SBCs was 13% in the whole group (Figure 1), 15,2% in females who got chest RT and 5,9% in those who didn’t (Figure 2). Two patients, both after chest RT, got bilateral SBCs. Seven of eight patients (with 9 SBCs) got chest RT with 24–80 (med. 36) Gy at the age of 12 to 18 (median 17) years. Four of them got ChT as well, all with AA, 3 with anthracyclines. One patient got one SBC after being treated with anthracyclines (cumulative dose of doxorubicin 300 mg/m^2^) and AA containing ChT without RT at the age of 13. We performed genetic testing in this patients and results were negative regarding genetic predisposition for breast cancer.

**Table 2:** Characteristics of ten SBCs in 8 patients.

	Range (median) in years
Age at diagnosis of HL	12–18 (16,5)
Age at diagnosis of SBC	28–52 (42)
Time from completion of HL therapy	14–39 (24)
Follow-up time after SBC diagnosis	5–24 (11.5)
	Number
**Method of SBC detection (in 8 SBCs diagnosed in screening era)**	
Patient detection	1
Clinical exam	1
Screening mammogram	5
Screening MRI	1
**Laterality of SBC**	
Unilateral	6
Bilateral	2
**Histology of SBC**	
Ductal carcinoma *in situ*	2
IDC	8
**TNM stage**	
0	2
IA	6
IB	1
IIA	0
IIB	1*
**Hormone receptor status in 8 IDCs**	
Positive	7
Negative	1
**HER 2 status in 8 IDCs**	
Positive	7
Negative	1
**Therapy for SBC**	
Mastectomy	9
Breast conserving surgery + postoperative breast RT	1^#^
Hormonal treatment	6
ChT	1
Hormonal treatment + ChT	1
**Vital status of 8 patients**	
Alive	6
Dead	2 (not of SBC)

SBC, subsequent breast cancer; HL, Hodgkin’ lymphoma; IDC, invasive ductal carcinoma; ChT, chemotherapy.

* Patient, diagnosed before screening era.

^#^ Patient refused mastectomy.

Eight SBCs were invasive ductal cancer (all but one with positive hormonal receptors, negative HER receptor) with/without *in situ* component, two SBCs were intraductal carcinomas *in situ*. Six invasive cancers were of stage T1N0, one of stage T1N1mi and one, diagnosed before era of screening, was of stage T2N1. Two of ten SBCs were diagnosed before era of breast screening, eight after introduction of screening program at LTFU clinic. Five SBCs were found on screening mammography, one by screening MRI, one was discovered during physical exam at regular LTFU and one by self-examination (in patient without chest RT). All patients but one were treated with mastectomy, six received hormonal treatment as well, one got ChT only, another one got ChT and hormonal treatment. None of them died of SBC.

## Discussion

To our knowledge, this is the first population based report on results of breast cancer screening in childhood HL survivors with more than 90% females at high-risk for SBC having regular breast cancer screening with long-term follow-up. Moreover, there are many reports on the benefit associated with early detection of SBC with screening in long-term cancer survivors after chest RT, but they are mostly hospital or multi institutional based with only part of patients being regularly screened for SBC (16, 18, 22–29). Furthermore, follow-up in some of these reports were short (25–27). Very few of these articles studied CCS only, even more rarely childhood HL survivors (18). Nevertheless, authors mostly confirmed that SBCs after chest RT detected by screening were more likely diagnosed at an earlier stage, were more frequently *in situ* carcinomas, smaller, without lymph node involvement and were more likely bilateral at diagnosis. Similarly, in current study all six invasive SBCs, diagnosed after 1999 when regular breast screening was introduced into our LTFU clinic, were of T1 stage and of N0, except in one with micrometastase in a sentinel node. Two SBCs were cancer *in situ*. Consequently, two patients only received ChT for their SBCs. None of females included in our cohort diagnosed with SBC died of breast cancer. Yeh et. al suggested with their models that annual screening with MRI (with or without mammography) starting at ages 25 to 30 years can avert half or more of the expected deaths from SBCs among young women previously exposed to chest radiation (30).

The cumulative incidence of SBCs in the whole cohort of our female pts 40 years after diagnosis of HL was 13% and 15,2% in those treated with chest RT. This incidence was relatively low in comparison with those reported in other studies, ranging from 12% to 26% by 25 to 30 years of follow-up (31–33). A possible explanation could be the fact that 75% of females treated with chest RT got AA containing ChT, which can lower the risk for SBC. Namely, toxic effect of AA on ovaries reduce exposure of radiation-damaged breast cells to stimulating effects of ovarian hormones (8, 34). On the other hand, only 8% of females treated with chest RT received pelvic RT.

SBCs in our cohort were histologically all ductal carcinoma, 2 *in situ*, eight invasive. Seven of eight invasive ductal carcinoma were estrogen and progesterone hormone positive, HER2 negative, one was triple negative. If we take into consideration only seven invasive SBCs detected in previously irradiated breast tissue, only one of seven (14%) was triple negative. Multiple authors reported that the characteristics of breast cancer developing after chest RT are biologically and pathologically similar to sporadic breast cancer (16, 25, 28, 29, 35). On the contrary, Horst reported that SBCs arising in previously irradiated breast tissue were more likely to be triple negative (i.e., estrogen and progesterone negative, HER2 negative) compared with age-matched sporadic invasive cancers (39% vs. 14%) and less likely to be HR positive (36). Similarly, the principal finding in the study of Demoor and coworkers was that invasive SBCs developing in previously irradiated breast tissue are frequently (29%) triple negative and very rarely HER2 positive (22). Interestingly, Castiglioni et al. in their case– control study reported a rate of 52% of triple negative SBCs if chest RT had been given 4 years or more after the menarche, vs. 6% if given before (37). Demoor at. al concluded that the proportion of triple negative phenotype SBC was higher in patients older at first cancer diagnosis (23). This observation can explain lower occurrence of triple negative SBCs in our series, because females in our cohort were younger at the time of chest RT than patients in the most above mention studies. Regarding hormone receptor expression in SBCs, authors of the CCS study reported that lower expression could be connected with toxic therapy for ovaries for HL (high dose of cyclophosphamide and RT that included ovaries) (34). The number of SBCs in our cohort is low and drawing significant conclusions in this regard is therefore impossible.

One of our patients got SBC at the age of 28, 15 years after treatment with chemotherapy without RT. Her SBC was invasive ductal carcinoma, hormone receptors positive, HER negative. Possible causative factor in this case could be high-dose of anthracyclines (over 250 mg/m^2^) as reported by Ehrhardt (18).

In the era of breast screening since 1999 onwards, five of seven SBCs in patients after chest RT were diagnosed by mammography, one with breast MRI and one was found by palpation during regular physical exam at the LTFU clinic. However, nowadays we know that dual imaging, breast MRI and mammography, provides a sensitive and specific approach to detect SBCs (18, 38) and the same is recommended in the international guidelines for breast cancer screening (21).

The proportion of CCS involved in population-based LTFU in Slovenia is high and from our experiences the most important thing to get survivors to LTFU is explaining patient at what risk is for certain somatic late sequelae and/or subsequent cancers according to previous cancer therapy. Only then, we can expect from survivors to come regularly to LTFU clinic for visits and to follow our recommendations according to international guidelines. We invest many efforts in teaching patients how to perform regular breast self-examination and empower them for breast screening. Yeazel's childhood cancer survival study demonstrated that cancer-screening practices among CCSs are below optimal levels (39). Only one third of the North American cohort of female CCS treated with chest RT had annual breast screening in the previous two years despite a guideline recommendation about annual breast imaging screening (40).

Limitation of our study is low number of patients in a cohort, but Slovenia is a small country with only 2 million inhabitants. Another weakness of the study is the fact that only two of eight females diagnosed with SBCs had genetic testing and results were negative regarding genetic predisposition for breast cancer in both.

## Conclusions

After introduction of regular breast screening in our female patients, who received chest RT in childhood, all SBCs were of early stage and no patients died of SBC. There was a low probability of SBC in the first 15 years after HL treatment completion. Survivors of pediatric HL should be informed about the risk of late sequelae of treatment for HL, including SBC. Regular follow-up with breast cancer screening and breast self-examination is of vital importance in those treated with chest RT.

## Data Availability

The raw data supporting the conclusions of this article will be made available by the authors, without undue reservation.

## References

[1] Robison LL Hudson MM. Survivors of childhood and adolescent cancer: life-long risks and responsibilities. Nat Rev Cancer. (2014) 14:61–70. 10.1038/nrc3634 24304873 PMC6425479

[2] Zadnik V Žagar T Tomšič S Lokar K Duratović Konjević A Zakotnik B. Preživetje bolnikov z rakom, zbolelih v letih 1997–2016 v sloveniji. Ljubljana: Onkološki inštitut Ljubljana (2020). 216 p. Available at: http://www.dlib.si/?URN=URN:NBN:SI:doc-Z8BOA2LC

[3] Oeffinger KC Mertens AC Sklar CA Kawashima T Hudson MM Meadows AT Chronic health conditions in adult survivors of childhood cancer. N Engl J Med. (2006) 355:1572–82. 10.1056/NEJMsa060185 17035650

[4] Olsen JH Möller T Anderson H Langmark F Sankila R Tryggvadóttír L Lifelong cancer incidence in 47,697 patients treated for childhood cancer in the nordic countries. J Natl Cancer Inst. (2009) 101:806–13. 10.1093/jnci/djp1046s 19470947

[5] Reulen RC Frobisher C Winter DL Kelly J Lancashire ER Stiller CA British Childhood cancer survivor study steering group. Long-term risks of subsequent primary neoplasms among survivors of childhood cancer. JAMA. (2011) 305:2311–19. 10.1001/jama.2011.747 21642683

[6] Mazic M C Reulen RC Jazbec J Zadravec Zaletel L. Trends in treatment of childhood cancer and subsequent primary neoplasm risk. Radiol Oncol. (2022) 56:380–9. 10.2478/raon-2022-0045 35848608 PMC9400439

[7] Bhatia S Robison LL Oberlin O Greenberg M Bunin G Fossati-Bellani F Breast cancer and other second neoplasms after childhood Hodgkin’s disease. N Engl J Med. (1996) 334:745–51. 10.1056/NEJM199603213341201 8592547

[8] Inskip PD Robison LL Stovall M Smith SA Hammond S Mertens AC Radiation dose and breast cancer risk in the childhood cancer survivor study. J Clin Oncol. (2009) 27:3901–7. 10.1200/JCO.2008.20.7738 19620485 PMC2734395

[9] Schellong G Riepenhausen M Ehlert K Brämswig J Dörffel W, German Working Group on the Long-Term Sequelae of Hodgkin's Disease, Schmutzler RK German Consortium for hereditary breast and ovarian cancer. Breast cancer in young women after treatment for Hodgkin's disease during childhood or adolescence–an observational study with up to 33-year follow-up. Dtsch Arztebl Int. (2014) 111:3–9. 10.3238/arztebl.2014.0003 24565270 PMC3948013

[10] Aleksandrova E Mihaylova I Sergieva S Parvanova V Ivanova D. Radiation-induced breast cancer in women with Hodgkin's disease. Rep Pract Oncol Radiother. (2014) 19:317–21. 10.1016/j.rpor.2014.01.003 25184056 PMC4150092

[11] McDonald AM Chen Y Wu J Hageman L Francisco L Kung M Total body irradiation and risk of breast cancer after blood or marrow transplantation: a blood or marrow transplantation survivor study report. J Clin Oncol. (2020) 38:2872–82. 10.1200/JCO.20.00231 32673169 PMC7460149

[12] van Leeuwen FE Klokman WJ Stovall M Dahler EC van’t Veer MB Noordijk EM Roles of radiation dose, chemotherapy, and hormonal factors in breast cancer following Hodgkin’s disease. J Natl Cancer Inst. (2003) 95:971–80. 10.1093/jnci/95.13.971 12837833

[13] Moskowitz CS Chou JF Wolden SL Bernstein JL Malhotra J Friedman DN Breast cancer after chest radiation therapy for childhood cancer. J Clin Oncol. (2014) 32:2217–23. 10.1200/JCO.2013.54.4601 24752044 PMC4100937

[14] Kuchenbaecker KB Hopper JL Barnes DR Phillips KA Mooij TM Roos-Blom MJ Risks of breast, ovarian, and contralateral breast cancer for BRCA1 and BRCA2 mutation carriers. JAMA. (2017) 317:2402–16. 10.1001/jama.2017.7112 28632866

[15] Begg CB Haile RW Borg A Malone KE Concannon P Thomas DC Variation of breast cancer risk among BRCA1/2 carriers. JAMA. (2008) 299:194–201. 10.1001/jama.2007.55-a 18182601 PMC2714486

[16] Henderson TO Amsterdam A Bhatia S Hudson MM Meadows AT Neglia JP Systematic review: surveillance for breast cancer in women treated with chest radiation for childhood, adolescent, or young adult cancer. Ann Intern Med. (2010) 152:444–55. 10.7326/0003-4819-152-7-201004060-00009 20368650 PMC2857928

[17] Henderson TO Moskowitz CS Chou JF Bradbury AR Neglia JP Dang CT Breast cancer risk in childhood cancer survivors without a history of chest radiotherapy: a report from the childhood cancer survivor study. J Clin Oncol. (2016) 34:910–8. 10.1200/JCO.2015.62.3314 26700127 PMC4871997

[18] Ehrhardt MJ Howell CR Hale K Baassiri MJ Rodriguez C Wilson CL Subsequent breast cancer in female childhood cancer survivors in the st jude lifetime cohort study (SJLIFE). J Clin Oncol. (2019) 37:1647–56. 10.1200/JCO.18.01099 31075046 PMC6804891

[19] Mulder RL Kremer LCM Hudson MM Bhatia S Landier W Levitt G Recommendations for breast cancer surveillance for female survivors of childhood, adolescent, and young adult cancer given chest radiation: a report from the international late effects of childhood cancer guideline harmonization group. Lancet Oncol. (2013) 14:621–9. 10.1016/S1470-2045(13)70303-6 PMC4257601 24275135

[20] Jereb B. Model for long-term follow-up of survivors of childhood cancer. Med Pediatr Oncol. (2000) 34:256–8. 10.1002/(sici)1096-911x(200004)34:4<256::aid-mpo5>3.0.co;2-8 10742062

[21] Mulder RL Hudson MM Bhatia S Landier W Levitt G Constine LS Updated breast cancer surveillance recommendations for female survivors of childhood, adolescent, and young adult cancer from the international guideline harmonization group. J Clin Oncol. (2020) 38:4194–207. 10.1200/JCO.20.00562 33078972 PMC7723685

[22] Demoor-Goldschmidt C Supiot S Oberlin O Helfre S Vigneron C Brillaud-Meflah V Clinical and diagnosis characteristics of breast cancers in women with a history of radiotherapy in the first 30 years of life: a French multicentre cohort study. Radiother Oncol. (2017) 124:200–3. 10.1016/j.radonc.2017.06.028 28733054

[23] Demoor-Goldschmidt C Supiot S Mahé MA Oberlin O Allodji R Haddy N. Clinical and histological features of second breast cancers following radiotherapy for childhood and young adult malignancy. Br J Radiol. (2018) 91:20170824. 10.1259/bjr.20170824 29493262 PMC6223287

[24] Elkin EB Klem ML Gonzales AM Ishill NM Hodgson D Ng AK Characteristics and outcomes of breast cancer in women with and without a history of radiation for Hodgkin’s lymphoma: a multi-institutional matched cohort study. J Clin Oncol. (2011) 29:2466–73. 10.1200/JCO.2010.32.4079 21576642 PMC3138631

[25] Diller L Medeiros Nancarrow C Shaffer K Matulonis U Mauch P Neuberg D Breast cancer screening in women previously treated for Hodgkin’s disease: a prospective cohort study. J Clin Oncol. (2002) 20:2085–91. 10.1200/JCO.2002.08.031 11956269

[26] Kwong A Hancock SL Bloom JR Pal S Birdwell RL Mariscal C Mammographic screening in women at increased risk of breast cancer after treatment of Hodgkin’s disease. Breast J. (2008) 14:39–48. 10.1111/j.1524-4741.2007.00524.x 18186864

[27] Lee L Pintilie M Hodgson DC Goss PE Crump M. Screening mammography for young women treated with supradiaphragmatic radiation for Hodgkin’s lymphoma. Ann Oncol. (2008) 19:62–7. 10.1093/annonc/mdm440 17878177

[28] Wolden SL Hancock SL Carlson RW Goffinet DR Jeffrey SS Hoppe RT. Management of breast cancer after Hodgkin’s disease. J Clin Oncol. (2000) 18:765–72. 10.1200/JCO.2000.18.4.765 10673517

[29] Dershaw DD Yahalom J Petrek JA. Breast carcinoma in women previously treated for Hodgkin disease: mammographic evaluation. Radiology. (1992) 184:421–3. 10.1148/radiology.184.2.1320281 1320281

[30] Yeh JM Lowry KP Schechter CB Diller LR Alagoz O Armstrong GT Clinical benefits, Harms, and cost-effectiveness of breast cancer screening for survivors of childhood cancer treated with chest radiation: a comparative modeling study. Ann Intern Med. (2020) 173:331–41. 10.7326/M19-3481 32628531 PMC7510774

[31] Bhatia S Yasui Y Robison LL Birch JM Bogue MK Diller L Late effects study group. High risk of subsequent neoplasms continues with extended follow-up of childhood Hodgkin’s disease: report from the late effects study group. J Clin Oncol. (2003) 21:4386–94. 10.1200/JCO.2003.11.059 14645429

[32] Kenney LB Yasui Y Inskip PD Hammond S Neglia JP Mertens AC Breast cancer after childhood cancer: a report from the childhood cancer survivor study. Ann Intern Med. (2004) 141:590–7. 10.7326/0003-4819-141-8-200410190-00006 15492338

[33] Wolden SL Lamborn KR Cleary SF Tate DJ Donaldson SS. Second cancers following pediatric Hodgkin’s disease. J Clin Oncol. (1998) 16:536–44. 10.1200/JCO.1998.16.2.536 9469338

[34] Moskowitz CS Chou JF Sklar CA Barnea D Ronckers CM Friedman DN Radiation-associated breast cancer and gonadal hormone exposure: a report from the childhood cancer survivor study. Br J Cancer. (2017) 117:290–9. 10.1038/bjc.2017.169 28632729 PMC5520518

[35] Ralleigh G. Screening for breast cancer in women with previous mantle radiotherapy for Hodgkin's disease. Breast Cancer Online. (2005) 8(10):E52. 10.1017/S1470903105003445

[36] Horst KC Hancock SL Ognibene G Chen C Advani RH Rosenberg SA Histologic subtypes of breast cancer following radiotherapy for Hodgkin lymphoma. Ann Oncol. (2014) 25:848–51. 10.1093/annonc/mdu017 24608191

[37] Castiglioni F Terenziani M Carcangiu ML Miliano R Aiello P Bertola L Radiation effects on development of HER2-positive breast carcinomas. Clin Cancer Res. (2007) 13:46–51. 10.1158/1078-0432.CCR-06-1490 17200337

[38] Hodgson DC Cotton C Crystal P Nathan PC. Impact of early breast cancer screening on mortality among young survivors of childhood Hodgkin’s lymphoma. J Natl Cancer Inst. (2016) 108(7):djw010. 10.1093/jnci/djw010 26933010

[39] Yeazel MW Oeffinger KC Gurney JG Mertens AC Hudson MM Emmons KM The cancer screening practices of adult survivors of childhood cancer: a report from the childhood cancer survivor study. Cancer. (2004) 100:631–40. 10.1002/cncr.20008 14745882

[40] Oeffinger KC Ford JS Moskowitz CS Diller LR Hudson MM Chou JF Breast cancer surveillance practices among women previously treated with chest radiation for a childhood cancer. JAMA. (2009) 301:404–14. 10.1001/jama.2008.1039 19176442 PMC2676434

